# Voluntary Attention Selectively Modulates Omission Responses

**DOI:** 10.1002/hbm.70518

**Published:** 2026-04-09

**Authors:** Tjerk T. Dercksen, Andreas Widmann, Nicole Wetzel

**Affiliations:** ^1^ Leibniz Institute for Neurobiology Magdeburg Germany; ^2^ Wilhelm Wundt Institute for Psychology Leipzig University Leipzig Germany; ^3^ Magdeburg‐Stendal University of Applied Sciences Stendal Germany

**Keywords:** attention, EEG, omission, precision weighting, predictive coding

## Abstract

Predictive coding conceptualizes attention as a weighting of prediction error signals. However, empirical findings on how attention influences common markers of prediction error have been inconsistent, likely because these markers are typically derived from stimulus‐evoked responses. To avoid stimulus‐related confounds and isolate effects related purely to prediction, we investigated how attention modulates brain responses to unexpected stimulus omissions. Using visual–auditory couplings where the auditory stimulus was occasionally omitted, we recorded EEG responses that revealed a multistage omission response—from early sensory to later higher‐level prediction error activity. Voluntary attention was manipulated along two dimensions: (1) toward the visual or auditory modality, and (2) toward the moment of stimulus presentation or sustained over time. Early sensory prediction error, reflected by the omission N1, was unaffected by any manipulation of attention. In contrast, later high‐level prediction error processing, reflected by omission P3 responses, was strongly affected by directing attention: robust responses were elicited when attention was directed to the auditory modality—where the prediction had been violated—but these were markedly reduced or absent when attention was directed to the visual modality. These results suggest an attentional system that does not affect low‐level sensory prediction error but is capable of influencing distinct stages in the processing hierarchy in service of task performance. This first investigation of how attention affects different stages of omission activity suggests that voluntary attention may modulate prediction error processing via specific neurotransmitter systems and demonstrates this approach's potential for reliably studying precision‐weighting in the brain.

## Key Points

- Unexpected auditory omissions elicited typical oN1, oN2, and oP3 responses in the EEG.
- Early omission activity (oN1) was unaffected by attention, whereas later responses (oP3) showed attention‐related modulation.
- Omission responses were stronger when attention was directed to the auditory modality than to the visual modality.

## Graphical abstract

Clapping sounds were occasionally omitted from a video (A). While early responses to omissions in the EEG showed similar amplitudes across conditions, larger responses were observed when attention was directed toward the auditory modality (D and E) compared to the visual modality (B and C).

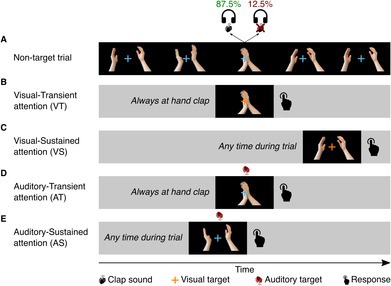

## 1 Introduction

On a daily basis, a considerable amount of conscious effort is devoted to directing attention toward or away from stimuli in our environment. Psychologically, such voluntary attention (also referred to as top‐down, endogenous, or goal‐driven attention) acts as a filter for our limited perceptual systems to support goal‐directed behavior (Broadbent 1958; Lavie 1995). Neuroscientific research increasingly reveals the complexity of this attentional system. Voluntary attention exerts its top‐down influences through both cortical–cortical (Miller and Buschman 2013) and cortical–subcortical (Wimmer et al. 2015) pathways, and engages multiple neuromodulatory (Thiele and Bellgrove 2018) and oscillatory (Xia et al. 2024) mechanisms. Nonetheless, the ultimate consequence of these processes appears largely convergent: an alteration of firing rates of neurons that represent attended versus unattended stimuli. The current study investigates how and at which stages this attention‐driven modulation of neural activity influences prediction error processing.

From a predictive processing perspective, alterations in firing rates due to attention are interpreted as an increase or decrease of the gain on prediction error units. In this framework, predictive processing (and related theories like predictive coding; Clark 2013; Friston 2005) assumes that the brain implements a generative model that continuously attempts to predict its sensory input. Discrepancies between predicted and actual input elicit prediction error responses, which in turn update perception and adapt the model to improve future predictions. Ideally, this updating relies on relevant prediction errors, which is why predictive processing conceptualizes attention as the weighting of prediction errors according to precision (Feldman and Friston 2010; Hohwy 2012, 2013).

While the concept of precision weighting offers an attractive explanation of attention within predictive processing, empirical support remains scarce. Especially, studies addressing voluntary attention in the auditory domain report small and sometimes conflicting effects (Heilbron and Chait 2018). Most of these investigations focus on the mismatch negativity (MMN). In such paradigms, an occasional “oddball” sound is presented within a stream of standard sounds to elicit a prediction error response, typically measured using EEG. This prediction error is assumed to be reflected in the MMN component, and some studies have reported enhanced MMN amplitudes when attention is directed toward sounds (Auksztulewicz and Friston 2015; Sussman 2007). However, debate continues over whether such effects should be attributed to deviance or to standard processing, and neglecting this distinction has likely contributed to inconsistent results in the past (Sussman 2007). One of the main complicating factors is that standards are sensitive to adaptation effects (Garrido et al. 2009), which are themselves subject to top‐down influences (Todorovic et al. 2011), making it difficult to isolate the genuine effect of attention on prediction error. Moreover, Schröger et al. (2015) point out that the MMN reflects a mixture of two signals: one related to unexpected input and another related to predicted but omitted input. Therefore, when interpreting attentional effects on the MMN, it remains unclear whether these reflect a specific gain on prediction error units associated with the standard stimulus or instead a more general amplification of exogenous auditory activity.

For these reasons, an approach might be preferred that circumvents confounds related to adaptation and is better able to isolate specific prediction effects. This can be achieved by analyzing brain responses not to unpredicted stimuli, but instead to unpredicted stimulus omissions. In such designs, a stimulus prediction is first established and then occasionally violated by omitting the stimulus. Theoretically, the presence of a prediction but absence of corresponding input should elicit a prediction error response. Crucially, this response presumably reflects only the prediction error related to the omitted stimulus and is not confounded by simultaneous bottom‐up activity (Braga and Schönwiesner 2022).

Only a few studies have investigated the influence of attention on omission responses, generally reporting increased amplitudes when attention is directed toward the auditory modality (Chennu et al. 2016; Hughes et al. 2001; Raij et al. 1997). However, these studies employed omission designs similar to oddball paradigms, in which deviant stimuli were replaced by omissions. Such designs require participants to form highly precise temporal predictions of when the omitted stimulus should occur; otherwise, temporally shifted responses may cancel out in the EEG (Hughes et al. 2001). The observed attention effects may therefore merely reflect interference with this timing process. In other cases, omission trials were also designated as targets (Chennu et al. 2016), which may have elicited target‐related rather than prediction‐related activity.

A major development in omission research over the past decades is the finding that introducing a time‐locking mechanism produces a sharper omission morphology with a consistent response pattern. Such mechanisms typically involve coupling a stimulus to either an action or a visual cue. For example, van Laarhoven et al. (2017) presented two hands on a screen that clap repeatedly. In most trials, the clap was accompanied by a sound, but occasionally the sound was omitted. These unexpected omissions were contrasted with a control condition in which the clap sound was never presented and therefore no prediction error was present. Subtracting the control condition filters out neural activity related to watching the clap and isolates the prediction error associated with the unexpected auditory omission.

Across paradigms, whether using visual–auditory (Stekelenburg and Vroomen 2015; van Laarhoven et al. 2017, van Laarhoven et al. 2020), motor‐auditory (Dercksen et al. 2020, 2022; Korka et al. 2020; SanMiguel, Saupe, et al. 2013; SanMiguel, Widmann, et al. 2013; Tast et al. 2024, 2025), or motor‐somatosensory (Dercksen et al. 2023) couplings, a recurring pattern of omission‐related ERP activity emerges. The earliest component, the oN1, is a negativity around 100 ms and is interpreted as the initial prediction error resulting from comparing the stimulus prediction template in sensory cortices to the actual (and unexpectedly absent) input (SanMiguel, Widmann, et al. 2013). The subsequent oN2 appears frontally around 170 ms and possibly reflects frontal activations to prediction error similar to those seen in the MMN (Dercksen et al. 2020). Finally, a sequence of oP3 components emerges from around 300 ms onward. Similar to stimulus‐evoked P300 responses, these likely reflect higher‐level contextual updating processes thought to be mediated by locus coeruleus (LC) activation (Dercksen et al. 2024; SanMiguel, Saupe, et al. 2013).

Given the limitations of earlier studies, the present EEG study set out to clarify how voluntary attention shapes auditory omission responses. We addressed this question using a version of the visual–auditory hand‐clapping paradigm, originally developed by Stekelenburg and Vroomen (2015) and replicated in several subsequent studies (van Laarhoven et al. 2017, 2020). We adapted this paradigm to systematically manipulate voluntary attention across modalities and time, while keeping stimulus predictions constant. The first and main manipulation directed participants' voluntary attention either to the auditory or the visual modality. If attention indeed increases the gain on prediction error units, responses to sound omissions should be larger when attention is directed to the auditory modality than when directed to the visual modality. The second manipulation examined whether this effect interacts with attention focused toward a specific moment in time: attention was either temporally focused on the moment of the hand clap or sustained throughout the trial. This allowed us to test for a potential role of transient/temporal attention (Seibold et al. 2023) in prediction error processing, where transient attention would potentially result in a stronger amplification of prediction error responses compared to sustained and therefore more distributed attention. Together, these factors resulted in a 2 × 2 design consisting of four experimental conditions: visual‐transient (VT), visual‐sustained (VS), auditory‐transient (AT), and auditory‐sustained (AS). Using this setup, we aimed to determine both (1) whether attention enhances the gain on prediction error units and (2) at which stages of prediction error processing (oN1, oN2, oP3) such effects occur.

## 2 Materials and Methods

### 2.1 Participants

EEG and behavioral data were acquired from 36 adult participants. Three participants were excluded because of large drifts in at least one of the control blocks, bringing the total participant number to 33 (17 females; age range = 18–36; mean age = 25.6 years, SD = 4.6 years). Participants gave written consent prior to the experiment and were compensated either financially or in the form of course credit points. The project was approved by the local ethics committee.

### 2.2 Stimuli

The visual stimuli consisted of a black background featuring a video of two hands performing a clapping motion, along with a central fixation cross. Stimuli were presented approximately 60 cm from participants' eyes. The hand‐clapping video consisted of 51 frames displayed at 60 frames/s (total duration: 0.85 s). The hands (Caucasian skin tone) started in a separated position, met at the center of the screen for a clap at frame 14 (0.23 s after movement onset), and ended again in a separated position similar to the starting position. For the remainder of the trial duration, the hands remained paused in the separated ending position. In the separated position, hands covered 14° horizontal and 12° vertical visual angle as measured from their extreme edges. The hands were aligned so that the clap occurred exactly at the location of the fixation cross. The fixation cross (0.67° × 0.67° visual angle) was displayed in colors from the Color‐Safe Palette (Mol 2018), specifically “sky blue” for the fixed standard cross and “orange” for the occasional target cross. The colors were adjusted to achieve approximate objective isoluminance. In case of a target cross presentation, the standard cross was replaced by the target cross for a duration of 0.2 s at the identical location, after which the standard cross was again presented.

A short (0.1 s) clapping sound was presented simultaneously with the visual clap (due to the use of headphones, a slight timing adjustment was introduced to account for the sound travel time that would naturally occur). The target sound was identical to the standard clapping sound, except played in reverse. This ensured that frequency characteristics were similar between standard and target sounds, preventing potential shifts from attention away from the frequencies of the standard clap. Sounds were presented at 70.4 dB SPL for all participants.

The duration difference between the visual and auditory targets was chosen on the basis of a pilot study, where these durations resulted in similar difficulties between the visual and auditory detection tasks.

### 2.3 Apparatus

Participants were seated in a dimly lit, electrically shielded and acoustically attenuated chamber, while EEG was continuously recorded. The experiment was programmed using Psychtoolbox (version 3.0.15; Brainard and Vision 1997) and ran on a Linux‐based system using GNU Octave (version 6.4.0). Auditory stimuli were presented using Sennheiser HD‐25 headphones. Visual stimuli were presented using a VIEWPixx‐EEG display (resolution 1920 horizontal × 1080 vertical—23.6 in. diagonal display size). To record target responses, a custom‐built infrared photoelectric button was used that was connected to the stimulus computer using an RTbox (Li et al. 2010). Participants' heads were stabilized using a fixed chin rest throughout all blocks to promote consistent visual responses.

### 2.4 Task and Procedure

Participants sat in front of a screen with their right index finger on a response button, watching a repeating video of a hand clap while fixating on a central cross. To fix the viewing angle across experimental blocks, participants placed their head on a chinrest that was adjusted to a comfortable height before the experiment started. The task of the participant was to press the button as quickly as possible in response to an occasionally presented target.

**FIGURE 1:**
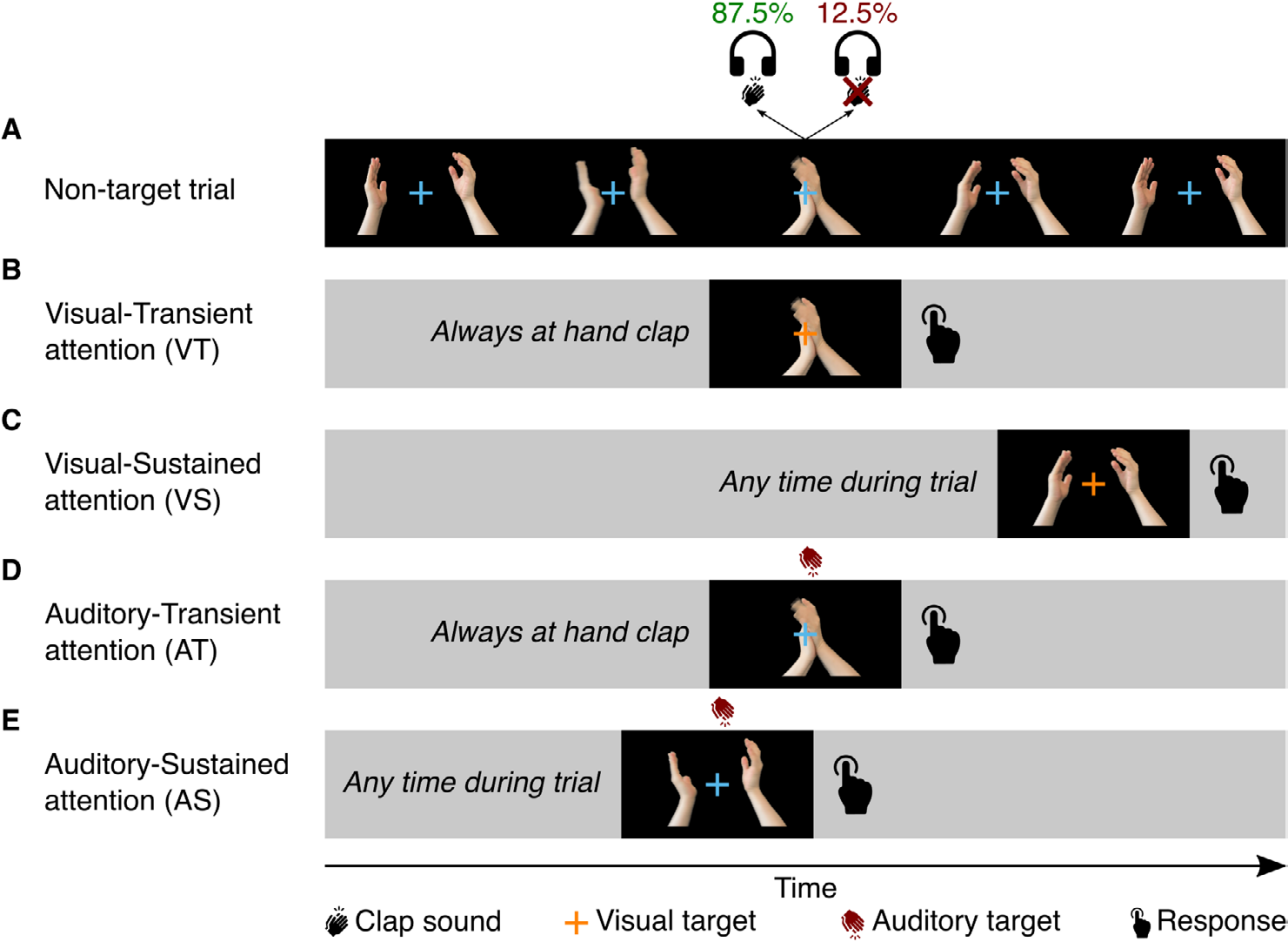
Representation of experimental design. (A) Typical, nontarget trial proceeding over time. At the moment of the hand clap, a clap sound is presented most of the time (87.5%), but occasionally omitted (12.5%). (B) Target in the VT condition, where at the moment of the hand clap, the fixation cross shortly turns orange. (C) Target in the VS condition, where at any moment during the trial, the fixation cross could shortly turn orange. (D) Target in the AT condition, where at the moment of the hand clap, an auditory target could be presented instead of the clapping sound. (E) Target in the AS condition, where at any moment during the trial, an auditory target could be presented. Participants had to respond to targets with a button press as fast as possible. In the corresponding control conditions, targets remained the same but standard sounds were omitted 100% of the time.

Claps were presented every 0.9–1.15 s. In most trials (87.5%) a sound was presented during the clap, while occasionally (12.5%) the sound was unexpectedly omitted (Figure 1a).

The experiment manipulated two types of attention: first, attention could be focused on either the visual or auditory modality. Second, the visual or auditory attention could be focused to a specific moment in time (transient attention) or be continuous (sustained attention). This 2 × 2 design resulted in a total of four experimental conditions: VT, VS, AT, and AS. To achieve the desired direction of attention, the conditions included an occasional target that had to be responded to using a rapid button press response upon detection. In the visual conditions (VT and VS), the target was a brief color change of the fixation cross from blue to orange (Figure 1b,c). In the auditory conditions (AT and AS), the target was a reversed hand clap sound (Figure 1d,e). In the transient attention conditions (VT and AT), targets could only occur precisely at the time of the hand clap (Figure 1b,d; replacing the hand clap sound in case of the auditory target). In the sustained attention conditions (VS and AS), targets could occur at any time during the trial (Figure 1c,e). Finally, to be able to subtract the neural activity related to the viewing of the clapping hands, each experimental condition had a corresponding control condition in which all standard hand clap sounds were omitted, while the targets remained the same.

Each of the four conditions was presented in three experimental blocks and had a corresponding control block, resulting in a total of 16 blocks across the experiment. To minimize switching costs, blocks belonging to the same condition were grouped together. The order of conditions was randomized across participants. Additionally, two of the control blocks were presented before the corresponding experimental blocks, and two after, with the order counterbalanced across participants (i.e., every condition had an identical amount of control blocks presented before and after the experimental condition).

The experimental blocks presented 210 standard clap trials and 30 unexpected omission trials (12.5%). Of the standard clap trials, 17 included a visual or auditory target (7.1%; replacing the clap in the AT condition). This resulted in a total of 630 standard clap trials (51 targets) and 90 unexpected omission trials per condition. The control blocks each presented 155 expected omission trials (or silent trials, as they are the standard) from which 11 were target trials (7.1%). Omission trials in the experimental and control blocks presented physically identical stimulation. Experimental blocks were around 4 min long and control blocks around 2.5 min. Total experiment time was roughly 80 min including breaks.

Omissions in the experimental blocks were randomly placed, under the restricting conditions that the first five trials of every block were always standard clap trials, and every two trials following an omission were never omission trials.

At the start of the experiment, participants completed a short training block. Instructions were presented on‐screen explaining the task and how attention should be directed. To optimize the attention manipulation, participants were explicitly instructed to respond to the targets as quickly as possible. Following the instructions, 10 practice trials for each condition were presented, each containing two targets. Once participants indicated they understood the task, the main experiment began.

Before each block, a reminder screen displayed the task instructions and specified whether the block was an experimental or control block (in control blocks, instructions stated “There will be no clapping sounds in this block”). After each block, a feedback screen showed participants their fastest and average reaction times, and the number of correctly detected targets.

At the start of each new condition, participants were shown an additional example block of 10 trials (including two targets) to familiarize themselves with the upcoming task. When a condition was completed, four questions appeared on the screen asking for participants' subjective experience of the condition:

1. How much of your attention was directed toward what you saw? (1 = not at all, 10 = completely);
2. How much of your attention was directed toward what you heard? (1 = not at all, 10 = completely);
3. How difficult was the task for you? (1 = not at all, 10 = extremely difficult);
4. Did you notice anything else?

### 2.5 Data Recording

EEG was recorded from a total of 63 active electrodes, placed according to the extended international 10–10 system at the following positions: Fp1/2, AFz, AF3/4, AF7/8, Fz, F1/2, F3/4, F5/6, F7/8, FC1/2, FC3/4, FC5/6, FT7/8, Cz, C1/2, C3/4, C5/6, T7/8, CPz, CP1/2, CP3/4, CP5/6, TP7/8, Pz, P3/4, P7/8, P1/2, P5/6, POz, PO3/4, PO7/8, O1/2, and the left (M1) and right (M2) mastoids. Furthermore, EOG was recorded from three electrodes placed left and right of the outer canthi of the eyes and below the left eye. The reference electrode was placed on the tip of the nose. An Actichamp amplifier (BrainProducts, Gilching, Germany) was used, recording at 500 Hz, DC‐coupled and with a 140 Hz low‐pass filter using BrainVision Recorder software (version 1.21).

### 2.6 EEG Data Preprocessing

EEG data analysis was performed with MATLAB software using the EEGLAB toolbox (Delorme and Makeig 2004). Data were filtered offline with a 0.1 Hz high‐pass filter (−6 dB, Kaiser windowed sinc FIR filter, order = 8024, *β* = 5, transition bandwidth = 0.2 Hz) and a 48 Hz low‐pass filter (−6 dB, Kaiser windowed sinc FIR filter, order = 402, *β* = 5, transition bandwidth = 4 Hz; this low‐pass filter has full attenuation at 50 Hz power line frequency). Data were segmented into epochs starting 300 ms before and ending 500 ms after stimulus/omission onset. Noisy channels were removed from the data, which were defined as having a robust *z*‐score of the robust standard deviation (0.7413 times the interquartile range) larger than 3 (Bigdely‐Shamlo et al. 2015). These channels were removed from analysis and interpolated after independent component analysis (ICA). Epochs exceeding a 500 μV signal‐change per epoch threshold were removed (in order to remove large nonstereotypical artifacts but to keep stereotypical artifacts as blinks and eye movements to be later removed using ICA). ICA was performed to correct for artifacts. This was done on data which were 1 Hz high‐pass filtered (−6 dB, Kaiser, order = 1604, *β* = 5, transition bandwidth = 1 Hz) and 48 Hz low‐pass filtered (same as above), as 1–2 Hz high‐pass filters improve ICA performance (Klug and Gramann 2021). Epoching and channel and trial removal were identical to the 0.1 Hz filtered dataset. After ICA, the obtained demixing matrix was subsequently applied to the 0.1–48 Hz filtered data. Artifact independent components (ICs) were judged by a human rater, aiming to remove all eye‐, heart‐, and muscle‐related components with support of the IClabel plugin (Pion‐Tonachini et al. 2019). On average, 14 components were rejected per participant (median = 14, min/max = 9/21, SD = 3). Each epoch was baseline corrected by subtracting the mean amplitude of the −300 to −200 ms window preceding stimulus onset. This interval was chosen as the typical −200 to –100 interval used in similar studies included sharp, likely visual processing related ERP activity. The first five trials of each block and the two trials following unexpected omissions were excluded from analysis to prevent confounding activity unrelated to the stimulus. Finally, trials that exceeded 120 μV signal‐change per epoch were excluded from analysis. In total, on average, 3337 trials were left per participant after preprocessing (median = 3368, min/max = 2938/3419, SD = 98).

Condition‐specific ERPs were computed for each participant. To analyze omission responses, the control condition ERPs were subtracted from the unexpected omission ERPs of their respective experimental condition. If time‐locked neural activity is present in addition to the control condition (reflecting the neural activity related to watching the clapping hands), this activity is considered to be prediction related.

### 2.7 Principal Component Analysis (PCA) of ERPs

Temporal PCA was used to analyze the grand‐average ERP omission data. This method aims to statistically decompose ERP waveforms into the constituent components of the resulting waveform, offering a data‐driven approach to ERP analysis (see Dien (2012) or Scharf et al. (2022) for tutorial treatments). PCA focused on the analysis of omission components (oN1, oN2, oP3) and was computed on the individual averages of the unexpected omissions in the VT, VS, AT, and AS conditions that had their control condition subtracted. Promax rotation (*κ* = 3) was used with a covariance relationship matrix and Kaiser weighting. The number of components to be retained was determined using Horn's parallel test.

For statistical testing of the components, distinct ROIs were determined based on earlier omission research (Dercksen et al. 2020, 2022, 2024; Korka et al. 2020; van Laarhoven et al. 2017, 2020; SanMiguel, Saupe, et al. 2013; SanMiguel, Widmann, et al. 2013; Stekelenburg and Vroomen 2015) in combination with the observed component topographies. The oN1 has consistently been measured over temporal electrodes, with occasional inclusion of fronto‐temporal electrodes (e.g., van Laarhoven et al. 2017; SanMiguel, Saupe, et al. 2013). Both early and late oN1 showed predominantly temporal activations. For early oN1, included electrodes were: T7, T8, C5, C6, C3, C4. For late oN1, included electrodes were: T7, T8, C5, C6. For the oN2, electrode selection is less clear, with some studies including frontal and others temporal regions. To not miss potential effects, two regions were defined. One was based on the predominantly temporal activation of the oN2 component, including electrodes: TP7, TP8, T7, T8, C5, C6, CP5, CP6, FT7, FT8, FC5, FC6. The other was based on literature reporting frontal oN2 activation (e.g., Dercksen et al. 2020, 2024), including electrode: Fz. The oP3 has consistently been measured over frontal, central, and parietal electrodes (e.g., Dercksen et al. 2020). All oP3 components showed predominantly fronto‐central activations. For oP3‐1, included electrodes were: Cz, Fz, FC1, FC2. For oP3‐2, included electrodes were: Fz, FC1, FC2. For oP3‐3, included electrodes were: Cz, Fz, FC1, FC2, F1, F2. Main results regarding oN1, temporal oN2, and oP3 were robust to reasonable variations in electrode selections.

For informational purposes, the ERPs of the auditory responses (i.e., standard hand clap trials minus their respective control conditions) and the ERPs of the control conditions are displayed in Figure 6. These were not subjected to further statistical analysis.

### 2.8 Statistical Analyses

Statistical testing was done using both frequentist and corresponding Bayesian statistics. This way, readers familiar with Bayesian statistics can benefit from its advantages (Rouder et al. 2009; Wagenmakers 2007) while still keeping results interpretable for readers more familiar with frequentist statistics. The main objective of the statistical analysis of the PCA was to determine whether components differ between attention conditions. This was done using a frequentist and Bayesian repeated‐measures ANOVA (rANOVA) including the factors Target Modality (visual vs. auditory) and Target Timing (transient vs. sustained). Additionally, omission components are only defined as such when they differ significantly from the control condition. As experimental conditions had the control conditions subtracted before PCA, this was tested by a frequentist and Bayesian one‐sample *t*‐test of the condition average against 0.

To analyze reaction times to targets, data were pooled across experimental and respective control conditions. Following that, a frequentist and Bayesian rANOVA including the factors Target Modality and Target Timing was performed.

To analyze target‐detection sensitivity, data were pooled across experimental and respective control conditions and analyzed using signal‐detection theory. For each subject and condition, hit rates were derived from target‐present trials and false‐alarm rates from non‐target trials. Rates were log‐linearly corrected (+0.5)/(+1) to avoid infinite *z*‐scores, and *d'* was computed as 𝑑′ = *Φ* ^−1^(HR) − *Φ* ^−1^(FAR). Following that, a frequentist and Bayesian rANOVA including the factors Target Modality and Target Timing was performed.

To analyze subjective attention ratings, 1–10 ratings to the first two questions (attention directed toward what was seen and what was heard) were entered into a single frequentist and Bayesian rANOVA. To directly assess the effectiveness of the attention manipulation while allowing for potential asymmetries between sensory modalities, ratings were recoded according to task relevance (such that ratings referring to the instructed modality were classified as attended and ratings referring to the non‐instructed modality as unattended), yielding the factor Attention (attended vs. unattended). The rANOVA further included the factors Target Modality (visual vs. auditory) and Target Timing (transient vs. sustained). This approach allowed a direct test of whether attention was successfully allocated to the instructed modality, as well as whether the magnitude of attentional allocation differed as a function of target modality or timing.

To analyze task difficulty ratings, 1–10 ratings to the third question were analyzed using a frequentist and Bayesian rANOVA including the factors Target Modality (visual vs. auditory) and Target Timing (transient vs. sustained).

Statistical testing was done using JASP (version 0.95.0; JASP Team 2021). For Bayesian rANOVAs, the JASP default fixed effects priors and random effects priors were used, defined as, respectively, *r* = 0.5 and *r* = 1. Bayesian rANOVAs tested all alternative models (main effects and interactions) against the null model, which included subjects and random slopes. The *BF*_inclusion_ factor (*BF*_incl_) across matched models was calculated for all variables to determine the evidence provided by the data for an effect if comparing all matched models including versus excluding the effect. For Bayesian one‐sample *t*‐tests, the null hypothesis was defined as a Cauchy prior distribution centered around 0 with a scaling factor of *r* = 0.707 (the default “medium” effect size prior scaling). Bayes Factor (*BF*_10_) was calculated using the JASP default number of sample repetitions and was interpreted following Lee and Wagenmakers (2013), who give the labels anecdotal (0.33–3), moderate (3–10 or 0.33–0.1), strong (10–30 or 0.1–0.033), and very strong (> 30 or < 0.033) for specific ranges of the Bayes Factor. We replaced the label “anecdotal” with “weak,” and “very strong” with “decisive” to aid interpretation. For frequentist *t*‐tests and rANOVAs, effect size was reported using Cohens *d* and the generalized *η* ^2^ (*η*_G_^2^; Bakeman 2005), respectively.

## 3 Results

All omission trials compared in this paradigm are physically exactly identical (a hand clap video where no hand clap sound is played). Omissions in the control conditions should contain the—attention modulated—neural activity related to the hand clap video free of any auditory prediction. Therefore, any additional activity left in the experimental conditions after subtracting the corresponding control condition can be considered prediction‐related activity. Subsequently, to determine the influence of voluntary attention on this prediction‐related activity, the controlled experimental conditions are compared directly. These main results are reported in Section 3.2, while Section 3.1 reports behavioral measures related to target reaction times and subjective experience, and in Section 3.3 stimulus‐related ERPs are displayed.

### 3.1 Behavior

Reaction times to targets in experimental conditions were 422 ms (SD = 123 ms) in VT, 400 ms (SD = 82 ms) in VS, 373 ms (SD = 95 ms) in AT, and 337 ms (SD = 53 ms) in AS. The rANOVA of reaction times favored the model including Target Modality and Target Timing (*BF*_10_ = 4.504 × 10^5^). Inclusion Bayes Factor provided decisive evidence for including Target Modality (*BF*_incl_ = 1.893 × 10^5^, *F*(1,32) = 55.37, *p* < 0.001, *η*_G_^2^ = 0.087), weak evidence for including Target Timing (*BF*_incl_ = 1.861, *F*(1,32) = 4.69, *p* = 0.038, *η*_G_^2^ = 0.026), and weak evidence for including the Target Modality × Target Timing interaction (*BF*_incl_ = 1.042, *F*(1,32) = 1.00, *p* = 0.324, *η*_G_^2^ = 0.001).

Participants' target‐detection sensitivity was near ceiling, demonstrating high hit rates and low false alarms across conditions for VT (*d′* = 4.85, SD = 1.051), VS (*d′* = 4.94, SD = 0.906), AT (*d′* = 4.81, SD = 0.868), and AS (*d′* = 4.80, SD = 0.906). The rANOVA of target‐detection sensitivity favored the null model. Inclusion Bayes Factor provided moderate evidence against including Target Modality (*BF*_incl_ = 0.222, *F*(1,32) = 1.05, *p* = 0.313, *η*_G_^2^ = 0.002), moderate evidence against including Target Timing (*BF*_incl_ = 0.171, *F*(1,32) = 0.187, *p* = 0.669, *η*_G_^2^ < 0.001), and strong evidence against including the Target Modality × Target Timing interaction (*BF*_incl_ = 0.052, *F*(1,32) = 0.189, *p* = 0.667, *η*_G_^2^ < 0.001).

**FIGURE 2:**
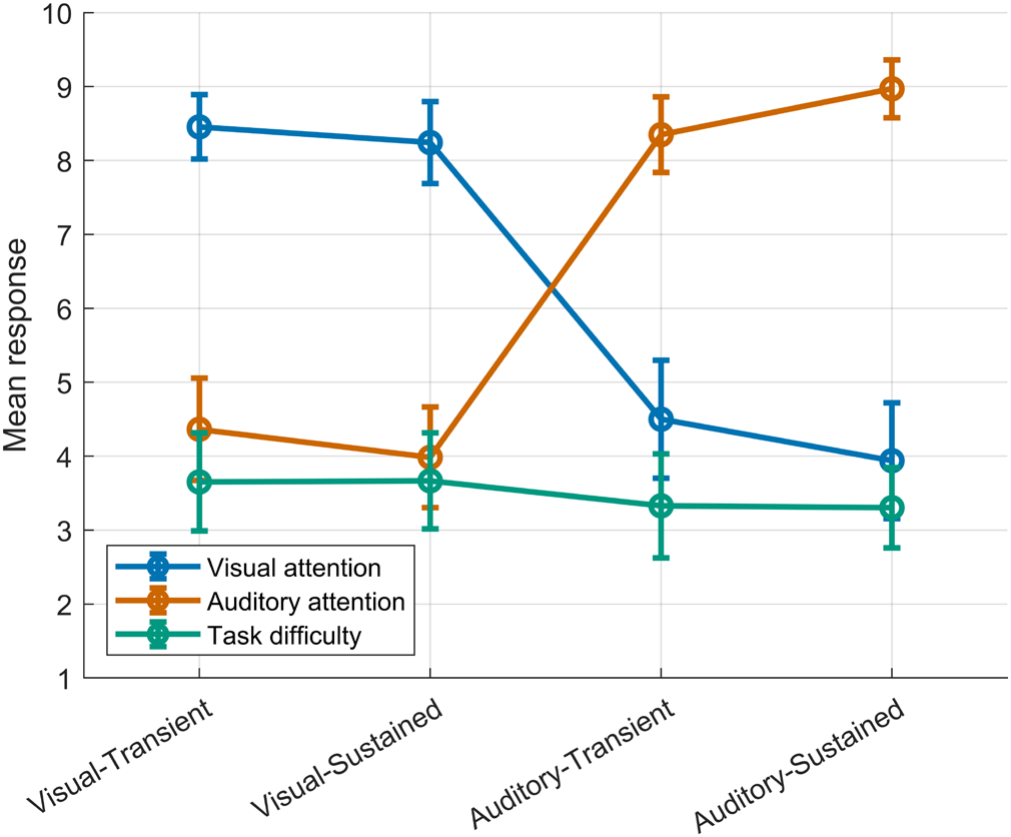
Subjective ratings in response to three questions regarding attention and task difficulty across the four conditions (*x* axis), indicating response ratings ranging from 1 to 10 (*y* axis). Question 1 (blue) asked how much attention was directed to what participants saw (visual attention). Question 2 (orange) asked how much attention was directed to what participants heard (auditory attention). Question 3 (green) asked how difficult the task was (task difficulty). Points represent mean ratings and 95% CIs.

Subjective indications of attention were in line with the experimental manipulation (Figure 2). Participants reported larger attention to what they saw (question one) in the VT (mean = 8.5, SD = 1.2) and VS (mean = 8.2, SD = 1.6) compared to the AT (mean = 4.5, SD = 2.3) and AS (mean = 3.9, SD = 2.2) conditions. Conversely, participants reported larger attention to what they heard (question two) in the AT (mean = 8.4, SD = 1.4) and AS (mean = 8.9, SD = 1.1) compared to the VT (mean = 4.4, SD = 1.9) and VS (mean = 4.0, SD = 1.9) conditions. The rANOVA of attention ratings favored the model including Attention (*BF*_10_ = 3.566 × 10^11^). Inclusion Bayes Factor provided decisive evidence for including Attention (*BF*_incl_ = 2.324 × 10^11^, *F*(1,32) = 171.25, *p* < 0.001, *η*_G_^2^ = 0.608), moderate evidence against including Target Modality (*BF*_incl_ = 0.169, *F*(1,32) = 0.98, *p* = 0.329, *η*_G_^2^ = 0.003), weak evidence against including Target Timing (*BF*_incl_ = 0.474, *F*(1,32) = 1.41, *p* = 0.244, *η*_G_^2^ = 0.001), weak evidence for including the Attention × Target Timing interaction (*BF*_incl_ = 1.698, *F*(1,32) = 6.72, *p* = 0.014, *η*_G_^2^ = 0.009), and at least moderate evidence against all other interaction effects (*BF*_incl_ < = 0.172). Follow‐up comparisons of the Attention × Target Timing interaction indicated that attention toward the unattended modality was rated slightly lower in the sustained compared to the transient attention condition (*BF*_10_ = 1.790, *t*(32) = 2.286, *p* = 0.029, *d* = 0.40), while the data rather provided weak evidence against a difference between sustained and transient condition attention ratings for the attended modality (*BF*_10_ = 0.582, *t*(32) = −1.592, *p* = 0.121, *d* = −0.28).

Difficulty was rated similarly across conditions for VT (mean = 3.7, SD = 1.9), VS (mean = 3.7, SD = 1.8), AT (mean = 3.3, SD = 2.0), and AS (mean = 3.3, SD = 1.5). This was confirmed by the rANOVA of question three which favored the null model. Inclusion Bayes Factor provided weak evidence against including Target Modality (*BF*_incl_ = 0.435, *F*(1,32) = 1.69, *p* = 0.203, *η*_G_^2^ = 0.008), moderate evidence against including Target Timing (*BF*_incl_ = 0.195, *F*(1,32) < 0.001, *p* = 1.000, *η*_G_^2^ < 0.001), and strong evidence against including the Target Modality × Target Timing interaction (*BF*_incl_ = 0.090, *F*(1,32) = 0.027, *p* = 0.872, *η*_G_^2^ < 0.001).

### 3.2 PCA of Omission Responses

PCA of the omission minus control ERPs extracted a total of 27 components (as determined by Horn's parallel test) explaining 94% of variance. Components generally demonstrated the expected pattern of auditory omission activity, showing oN1, oN2, and oP3 responses. Components were selected for further analysis based on their theoretical relevance based on earlier studies, resulting in 6 components: early oN1, late oN1, oN2, oP3‐1, oP3‐2, and oP3‐3. Characteristics of these components are summarized in Table 1. Components that were discarded for further analysis did not demonstrate meaningful differences between conditions.

**TABLE 1:** Overview of component characteristics.

Component name	Component number	Explained variance	Peak latency	Activation topography
Early oN1	8	4.1%	104 ms	Temporal
Late oN1	11	3.3%	136 ms	Temporal
oN2	4	5.7%	168 ms	Frontal/temporal
oP3‐1	6	4.4%	338 ms	Fronto‐central
oP3‐2	7	4.2%	370 ms	Fronto‐central
oP3‐3	1	18.3%	432 ms	Fronto‐central

#### 3.2.1 Early oN1

**FIGURE 3:**
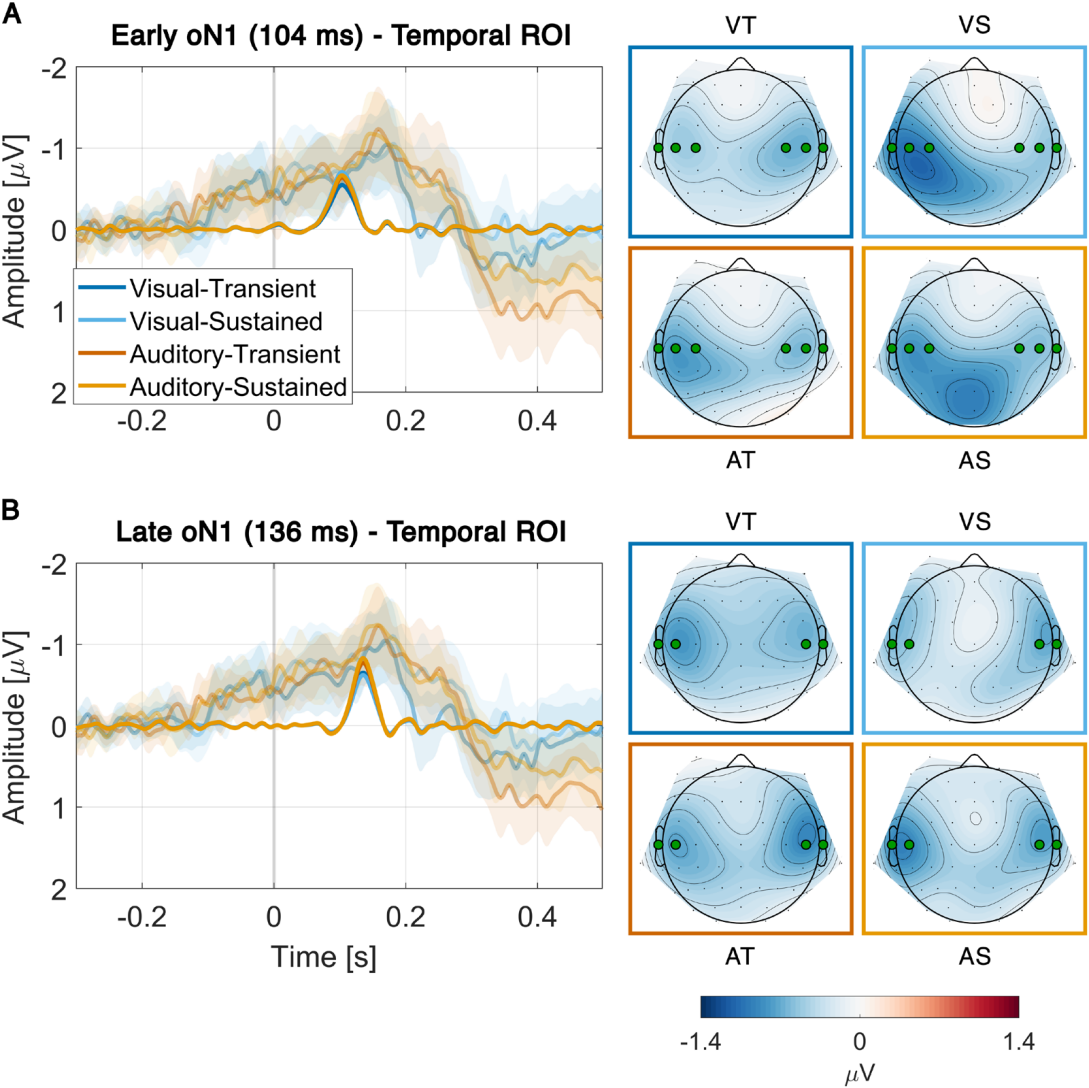
Early (A) and late (B) oN1 components. The left plot in each panel shows difference waves (experiment minus control), with time 0 marking the predicted onset of an omitted sound. Difference waves are shown for reconstructed PCA components (opaque) and grand‐average ERPs with 95% CIs (transparent). The right side in each panel shows the corresponding PCA component topographies with highlighted electrodes indicating ROIs.

Similar to earlier studies (Dercksen et al. 2022, 2024; Korka et al. 2020), PCA divided the first negative wave in multiple components. Analogous to the earlier findings, we refer to these components with early and late oN1, where “o” stands for omission and “N” for the polarity (negative). The early oN1 had a peak latency of 104 ms and was maximal over temporal leads (Figure 3a). Compared to the control conditions, the data provided moderate or strong evidence for elicitation of the early oN1 response for all conditions (VT: *BF*_10_ = 6.33, *t*(32) = 2.91, *p* < 0.01, *d* = 0.51; VS: *BF*_10_ = 32, *t*(32) = 3.62, *p* < 0.001, *d* = 0.63; AT: *BF*_10_ = 6.50, *t*(32) = 2.93, *p* < 0.01, *d* = 0.51; AS: *BF*_10_ = 45, *t*(32) = 3.76, *p* < 0.001, *d* = 0.66). The rANOVA favored the null model. Inclusion Bayes Factor provided moderate evidence against including Target Modality (*BF*_incl_ = 0.16, *F*(1,32) = 0.02, *p* = 0.89, *η*_G_^2^ < 0.001), moderate evidence against including Target Timing (*BF*_incl_ = 0.19, *F*(1,32) = 0.20, *p* = 0.66, *η*_G_^2^ = 0.002), and strong evidence against including the Target Modality × Target Timing interaction (*BF*_incl_ = 0.04, *F*(1,32) = 0.12, *p* = 0.73, *η*_G_^2^ < 0.001).

#### 3.2.2 Late oN1

The late oN1 had a peak latency of 136 ms and was maximal over temporal leads (Figure 3b). Compared to the control conditions, the data provided decisive evidence for elicitation of the late oN1 response for all conditions (VT: *BF*_10_ = 81, *t*(32) = 3.99, *p* < 0.001, *d* = 0.70; VS: *BF*_10_ = 48, *t*(32) = 3.78, *p* < 0.001, *d* = 0.66; AT: *BF*_10_ = 39, *t*(32) = 3.70, *p* < 0.001, *d* = 0.64; AS: *BF*_10_ = 168, *t*(32) = 4.28, *p* < 0.001, *d* = 0.75). The rANOVA favored the null model. Inclusion Bayes Factor provided moderate evidence against including Target Modality (*BF*_incl_ = 0.23, *F*(1,32) = 1.49, *p* = 0.23, *η*_G_^2^ = 0.006), moderate evidence against including Target Timing (*BF*_incl_ = 0.16, *F*(1,32) = 0.01, *p* = 0.93, *η*_G_^2^ < 0.001), and strong evidence against including the Target Modality × Target Timing interaction (*BF*_incl_ = 0.05, *F*(1,32) = 0.06, *p* = 0.80, *η*_G_^2^ < 0.001).

#### 3.2.3 oN2

**FIGURE 4:**
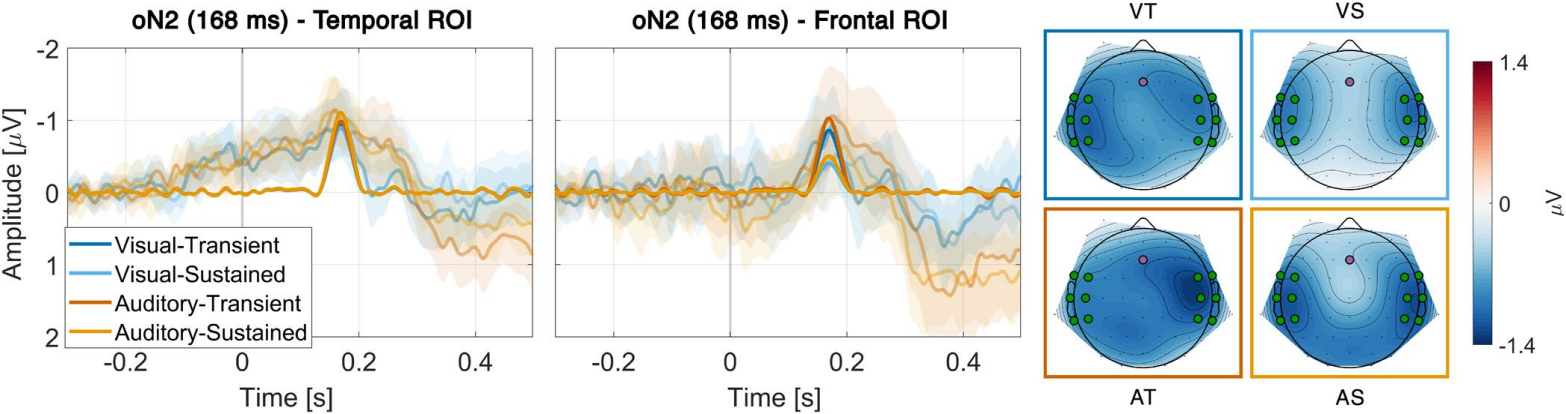
Temporal (left) and frontal (middle) oN2 difference waves and PCA component topographies (right), with highlighted electrodes indicating ROIs. For difference waves (experiment minus control), Time 0 marks the predicted onset of an omitted sound, showing reconstructed PCA components (opaque) and grand‐average ERPs with 95% CIs (transparent).

The oN2 had a peak latency of 168 ms and was maximal over temporal leads. However, since past findings typically locate oN2 over frontal leads, two ROIs were tested: temporal and frontal (Figure 4).

##### 3.2.3.1 Temporal

Compared to the control conditions, the data provided decisive evidence for elicitation of the oN2 over temporal electrodes for all conditions (VT: *BF*_10_ = 92, *t*(32) = 4.05, *p* < 0.001, *d* = 0.70; VS: *BF*_10_ = 154, *t*(32) = 4.25, *p* < 0.001, *d* = 0.74; AT: *BF*_10_ = 511, *t*(32) = 4.71, *p* < 0.001, *d* = 0.82; AS: *BF*_10_ = 1066, *t*(32) = 4.99, *p* < 0.001, *d* = 0.87). The rANOVA favored the null model. Inclusion Bayes Factor provided moderate evidence against including Target Modality (*BF*_incl_ = 0.19, *F*(1,32) = 0.79, *p* = 0.38, *η*_G_^2^ = 0.003), moderate evidence against including Target Timing (*BF*_incl_ = 0.18, *F*(1,32) = 0.03, *p* = 0.86, *η*_G_^2^ < 0.001), and strong evidence against including the Target Modality × Target Timing interaction (*BF*_incl_ = 0.05, *F*(1,32) = 0.18, *p* = 0.67, *η*_G_^2^ = 0.001).

##### 3.2.3.2 Frontal

Compared to the control conditions, the data provided moderate evidence for elicitation of the oN2 over frontal electrodes in the VT condition (*BF*_10_ = 8.24, *t*(32) = 3.03, *p* < 0.01, *d* = 0.53), weak evidence against elicitation in the VS condition (*BF*_10_ = 0.50, *t*(32) = 1.48, *p* = 0.149, *d* = 0.26), moderate evidence for elicitation in the AT condition (*BF*_10_ = 6.03, *t*(32) = 2.89, *p* < 0.01, *d* = 0.50), and weak evidence against elicitation in the AS condition (*BF*_10_ = 0.76, *t*(32) = 1.78, *p* = 0.085, *d* = 0.31). The rANOVA favored the null model. Inclusion Bayes Factor provided strong evidence against including Target Modality (*BF*_incl_ = 0.19, *F*(1,32) = 0.20, *p* = 0.66, *η*_G_^2^ = 0.002), weak evidence against including Target Timing (*BF*_incl_ = 0.45, *F*(1,32) = 2.52, *p* = 0.12, *η*_G_^2^ = 0.020), and strong evidence against including the Target Modality × Target Timing interaction (*BF*_incl_ = 0.09, *F*(1,32) = 0.02, *p* = 0.90, *η*_G_^2^ < 0.001).

#### 3.2.4 oP3‐1

**FIGURE 5:**
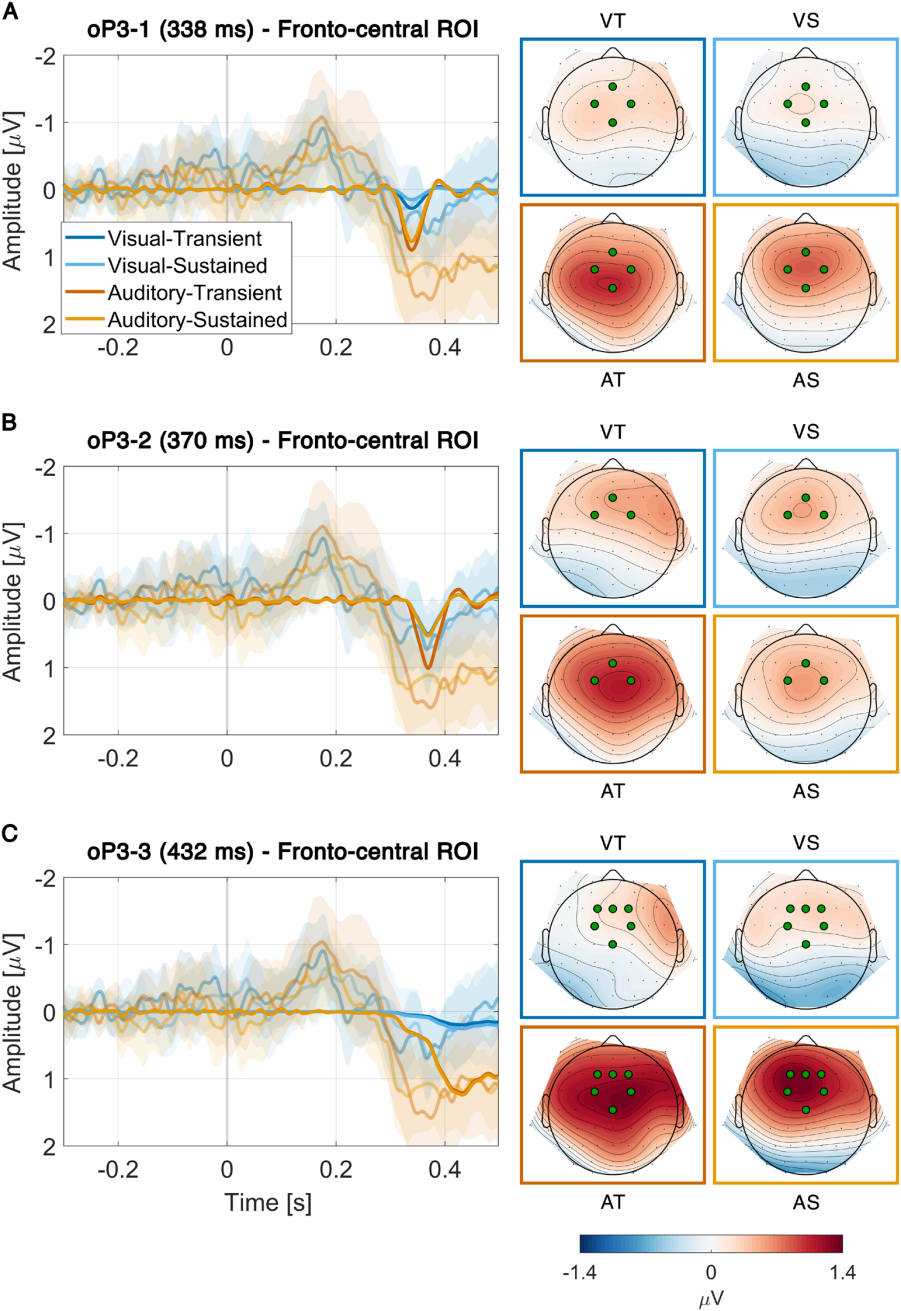
Later oP3 activity divided into oP3‐1 (A), oP3‐2 (B), and oP3‐3 (C) components. The left plot in each panel shows difference waves (experiment minus control), with Time 0 marking the predicted onset of an omitted sound. Difference waves are shown for reconstructed PCA components (opaque) and grand‐average ERPs with 95% CIs (transparent). The right side in each panel shows the corresponding PCA component topographies with highlighted electrodes indicating ROIs.

The oP3‐1 had a peak latency of 338 ms and was maximal over fronto‐central leads (Figure 5a). Compared to the control conditions, the data provided weak evidence against elicitation of the oP3‐1 in the VT condition (*BF*_10_ = 0.37, *t*(32) = 1.23, *p* = 0.23, *d* = 0.21), moderate evidence against elicitation in the VS condition (*BF*_10_ = 0.23, *t*(32) = 0.64, *p* = 0.53, *d* = 0.11), moderate evidence for elicitation in the AT condition (*BF*_10_ = 4.66, *t*(32) = 2.77, *p* < 0.01, *d* = 0.48), and moderate evidence for elicitation in the AS condition (*BF*_10_ = 9.80, *t*(32) = 3.11, *p* < 0.01, *d* = 0.54). The rANOVA favored the model including Target Modality (*BF*_10_ = 2.47). Inclusion Bayes Factor provided weak evidence for including Target Modality (*BF*_incl_ = 1.73, *F*(1,32) = 6.32, *p* = 0.017, *η*_G_^2^ = 0.042), moderate evidence against including Target Timing (*BF*_incl_ = 0.21, *F*(1,32) = 0.24, *p* = 0.63, *η*_G_^2^ = 0.002), and moderate evidence against including the Target Modality × Target Timing interaction (*BF*_incl_ = 0.14, *F*(1,32) < 0.001, *p* = 0.99, *η*_G_^2^ < 0.001).

#### 3.2.5 oP3‐2

The oP3‐2 had a peak latency of 370 ms and was maximal over fronto‐central leads (Figure 5b). Compared to the control conditions, the data provided weak or strong evidence for elicitation of the oP3‐2 for all conditions (VT: *BF*_10_ = 1.40, *t*(32) = 2.15, *p* = 0.039, *d* = 0.37; VS: *BF*_10_ = 2.95, *t*(32) = 2.55, *p* = 0.016, *d* = 0.44; AT: *BF*_10_ = 115, *t*(32) = 4.13, *p* < 0.001, *d* = 0.72; AS: *BF*_10_ = 1.67, *t*(32) = 2.25, *p* = 0.032, *d* = 0.39). The rANOVA favored the null model. Inclusion Bayes Factor provided weak evidence against including Target Modality (*BF*_incl_ = 0.39, *F*(1,32) = 1.20, *p* = 0.28, *η*_G_^2^ = 0.010), moderate evidence against including Target Timing (*BF*_incl_ = 0.30, *F*(1,32) = 1.69, *p* = 0.20, *η*_G_^2^ = 0.008), and moderate evidence against including the Target Modality × Target Timing interaction (*BF*_incl_ = 0.23, *F*(1,32) = 1.89, *p* = 0.18, *η*_G_^2^ = 0.009).

#### 3.2.6 oP3‐3

The oP3‐3 had a peak latency of 432 ms and was maximal over fronto‐central leads (Figure 5c). Compared to the control conditions, the data provided moderate evidence against elicitation of the oP3‐3 in the VT condition (*BF*_10_ = 0.24, *t*(32) = 0.75, *p* = 0.46, *d* = 0.13), moderate evidence against elicitation in the VS condition (*BF*_10_ = 0.25, *t*(32) = 0.82, *p* = 0.42, *d* = 0.14), moderate evidence for elicitation in the AT condition (*BF*_10_ = 4.01, *t*(32) = 2.70, *p* = 0.01, *d* = 0.47), and decisive evidence for elicitation in the AS condition (*BF*_10_ = 194, *t*(32) = 4.34, *p* < 0.001, *d* = 0.76). The rANOVA favored the model including Target Modality (*BF*_10_ = 5.20). Inclusion Bayes Factor provided moderate evidence for including Target Modality (*BF*_incl_ = 3.76, *F*(1,32) = 8.17, *p* < 0.01, *η*_G_^2^ = 0.065), moderate evidence against including Target Timing (*BF*_incl_ = 0.18, *F*(1,32) = 0.03, *p* = 0.87, *η*_G_^2^ < 0.001), and moderate evidence against including the Target Modality × Target Timing interaction (*BF*_incl_ = 0.16, *F*(1,32) < 0.001, *p* = 0.97, *η*_G_^2^ < 0.001).

### 3.3 Stimulus‐Related ERPs

**FIGURE 6:**
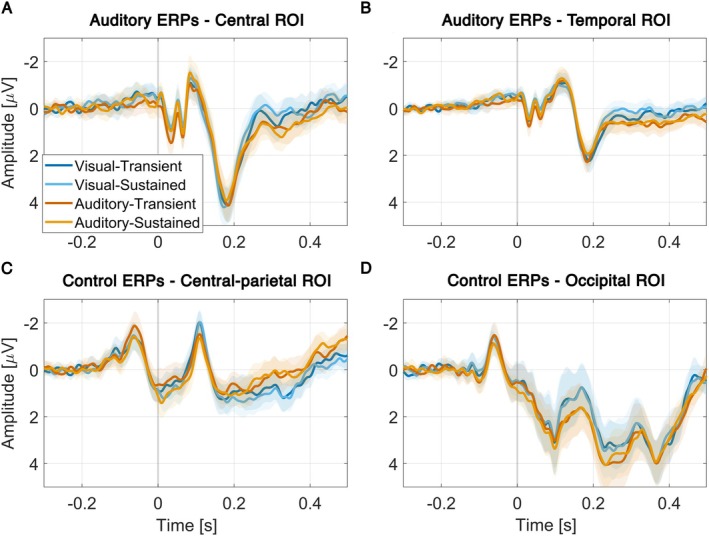
(A) Auditory responses are shown over central electrodes including channels Cz, FC1, FC2, C1, C2, CP1, CP2, and CPz, as well as over (B) temporal electrodes T7, T8, C5, C6. These plots have control activity subtracted. (C) Visual responses to the hand clap motion in the control conditions are shown over central‐parietal electrodes including channels Cz, C1, C2, CPz, as well as over (D) occipital electrodes O1 and O2. Time 0 marks the onset of the sound (experiment conditions) or expected omission (control conditions).

Stimulus‐related ERPs for auditory responses to the clapping sounds in the experimental conditions and visual responses in the control condition are shown in Figure 6. A detailed statistical analysis of these ERPs is outside the scope of this paper.

## 4 Discussion

The current study investigated whether, and at which levels, attention influences prediction error processing. To this end, we measured omission responses using visual–auditory couplings while manipulating attention. Specifically, voluntary attention was manipulated either toward the visual or auditory modality, and either focused toward the moment of stimulus presentation or sustained throughout the trial. Across all conditions, the expected pattern of omission responses was present, consisting of oN1, oN2, and oP3 components. Importantly, the effects of modality were restricted to the oP3 timeframe: when attention was directed to the modality where the omission occurred, that is, to the target clap sound, larger oP3 components were elicited, whereas attention to the visual targets either reduced or abolished oP3 components. In contrast, no modality effects were found for the oN1 or oN2, and no convincing differences emerged between transient and sustained attention. With subjective ratings indicating clear differences in attention allocation but comparable task difficulty (Figure 2), the observed ERP differences are highly likely to have been driven by the direction of voluntary attention.

### 4.1 No Effects of Attention on Early Prediction Error‐Related Activity (oN1)

In line with earlier studies using PCA to decompose omission waves (Dercksen et al. 2022; Korka et al. 2020; Tast et al. 2024, 2025), the oN1 was divided into early and late components (Figure 3a,b). The absence of attention effects for these components was somewhat unexpected. The sound‐related oN1 is thought to reflect the difference between the predicted sensory template in the auditory cortex and the actual auditory input. Predictive coding theories of attention (Feldman and Friston 2010) propose that the gain on prediction error units of this sensory template should increase when attention is directed to the auditory modality. Accordingly, components reflecting this sensory prediction error would be expected to show larger amplitudes, consistent with findings from a subset of MMN studies (Sussman 2007). Previous omission studies, using somewhat different designs, have likewise reported attentional modulations of early auditory omission activity (Chennu et al. (2016) around 170 ms; Raij et al. (1997) around 170 ms), although these were unlikely to reflect oN1 activity given their latency. However, as discussed in the introduction, the present paradigm is arguably better suited than MMN designs to control for confounding factors. Moreover, the diminished omission responses in earlier studies may have reflected imprecise temporal predictions rather than genuine attention effects. The current results suggest that when prediction is held constant and only attention is manipulated, the sensory component of omission responses remains unaffected, consistent with findings by Hughes et al. (2001). This adds to a growing body of work showing inconsistent attentional effects on prediction error (Heilbron and Chait 2018), but—unlike earlier studies—excludes several important confounding influences (such as those related to processing of standard stimuli in typical oddball paradigms).

The present results do not definitively rule out attentional influences on sensory prediction error. For example, one possibility is that precision weighting effects are not expressed as amplitude increases but rather as response sharpening—enhanced frequency selectivity of auditory neural populations under attention (Kauramäki et al. 2007; Okamoto et al. 2007). Such effects may be better captured using decoding approaches (Demarchi et al. 2019; Hauswald et al. 2024; Ishida et al. 2024), which could reveal attentional sharpening of omission responses in future studies. Alternatively, effects of attention on sensory prediction error could be limited to gamma frequencies (Bastos et al. 2012), which are known to be elicited by unexpected sound omissions (Todorovic et al. 2011) but were not examined in this study.

Another consideration is that attention to the auditory modality typically modulates the N1 component of sound‐evoked ERPs (Lange 2013). In our data, however, attention effects were mainly evident in visual responses (Figure 6c,d) rather than in auditory responses to the hand‐clap sounds (Figure 6a,b). This may be interpreted as an absence of precision weighting in the auditory cortex, which could account for the lack of oN1 modulation if omission activity is considered the mirror image of stimulus‐related predictions (Schröger et al. 2015). Despite efforts to match target and standard sound characteristics, participants in our experiment may have preferentially tuned attention to the features of the target sound. This would make standard sound features irrelevant to the target‐detection task, which in terms of predictive coding constitutes a decrease of attention (Mirza et al. 2019). As a result, prediction error units in sensory cortices representing the standard sound would not be upweighted, preventing amplification of the N1 and oN1 responses. Future work could address this by more strongly directing attention to the features of the omitted sound. However, it should be noted that in earlier omission studies, comparable methods were used to direct attention (such as counting deviant target sounds; Raij et al. 1997). The absence of early attention effects on omission activity may therefore also be interpreted as a signal that in earlier studies, differences in prediction might have been mistaken for attention effects.

### 4.2 Indications of oN2 Sensitivity to Transient Versus Sustained Attention

The oN2 typically presents as a frontal response, but in the current study it was predominantly temporal (Figure 4). This pattern might be specific to the visual–auditory paradigm, as a predominantly temporal oN2 has been reported before when using hand clap motions to elicit omission responses (van Laarhoven et al. 2017, 2020). Over frontal electrodes, the oN2 appeared only when attention was focused toward the moment of the hand clap rather than sustained, although a direct comparison did not yield significant differences. While this restricts drawing firm conclusions, the apparent dependence of the frontal oN2 on transient attention provides an additional data point for characterizing this component, which has generally been less reliable than the oN1 and oP3 (Korka et al. 2020).

### 4.3 Convincing Attention Effects at Later Processing Stages (oP3)

The main effects of attention in this study were confined to the oP3, starting around 330 ms after omission onset. This resembles the intracranial omission study of Hughes et al. (2001), who reported a later P3‐like effect only under attended conditions. More broadly, our results align with studies demonstrating that prediction and attention interact primarily at later, higher‐level rather than early sensory stages of processing (Alilović et al. 2019; Marzecová et al. 2017; Smout et al. 2019). Given its topographical similarity to the P3 (Dercksen et al. 2020; Baragona et al. 2025) and its relationship with pupil size changes (Dercksen et al. 2023), the oP3 is typically interpreted as reflecting processes similar to those underlying stimulus‐evoked P3 responses. Within a predictive coding framework, these responses are thought to represent model‐updating processes that revise the internal generative model (Wacongne et al. 2011). The fact that attention specifically modulates this activity opens several avenues for interpretation.

First, whereas earlier studies have consistently observed an association between stronger oN1/oN2 and subsequent oP3 activity (e.g., Dercksen et al. 2020), the present findings reveal a modulation largely confined to the oP3. This supports a view of attention as a sophisticated process capable of independently modulating gain at different stages of the processing hierarchy in service of task performance (Schröger et al. 2015). The P3/oP3 stage is typically associated with phasic activity of the LC‐NE system (Nieuwenhuis et al. 2005), although other systems such as dopamine may also contribute (Polich 2007). Accordingly, the distinct effects of voluntary attention observed here may be predominantly driven by norepinephrine‐mediated gain modulation (Thiele and Bellgrove 2018) in the cortex.

A second perspective concerns the absence of much of the oP3 when attention was directed away from sounds. In these conditions, oP3 activity was either diminished (Figure 5b) or absent altogether (Figure 5a,c). On one hand, this aligns with previously reported reductions of P3a (Chennu et al. 2013; Wetzel et al. 2006) and P3b (Polich 2003) amplitudes under diverted attention. On the other hand, components such as the P3a can remain robust—or even unchanged—when a deviant is outside the focus of attention but is particularly salient (Berti et al. 2004; Jones et al. 2013). Although the omission of a predicted stimulus arguably represents the most extreme form of prediction error for a particular stimulus, oP3 activity here was almost completely silenced. This calls for further investigation into what constitutes attention‐capturing salience (Baragona et al. 2025), particularly, the relative contributions of prediction errors elicited by difference signals (between predictions and actual input) versus purely feedforward input (Furutachi et al. 2024).

In studies using motor‐auditory couplings, PCA divided the oP3 into subcomponents that can be clearly distinguished by latency and topography, resembling stimulus‐evoked P3a, P3b, and novelty‐P3 (Dercksen et al. 2020, 2023; Korka et al. 2020). In the present study, however, topographies showed a similar fronto‐central topography between subcomponents, indicating possibly similar sources. This distinction between motor‐auditory and visual–auditory couplings had already been noted by Stekelenburg and Vroomen (2015) and appears to be confirmed by our PCA. That is, the typical oP3‐2 over parietal electrodes in motor‐auditory studies seems to be absent in visual–auditory couplings, and generally oP3 amplitudes were slightly diminished. Future work may determine whether attention also modulates the motor‐related parietal oP3 component and further clarify the functional distinction between motor‐ and visual‐related couplings.

Finally, the present findings may help to better understand the role of attention during learning. Voluntary attention has been shown to substantially increase learning rates in perceptual learning (Byers and Serences 2012; Nguyen et al. 2020). Although the neural mechanisms of perceptual learning remain incompletely understood, recent evidence points to a reweighting of superficial layers in sensory cortices (Jia et al. 2020, 2024). Interestingly, LC activity has been found to strongly promote such plasticity in animal models (Glennon et al. 2019; Jordan and Keller 2023; Poe et al. 2020). If the oP3 indeed predominantly reflects LC–NE activity, this reveals a potential pathway linking voluntary attention to enhanced learning. Specifically, the direction of voluntary attention increases LC–NE gain, thereby accelerating reweighting and tuning processes in sensory areas, resulting in superior learning (or model updating in the context of predictive processing). Given the broad role of the LC across the brain (Jordan 2024), this mechanism may generalize to other areas of learning as well.

### 4.4 Conclusions

Taken together, these findings indicate that neither modality‐specific nor temporally allocated voluntary attention influences elicitation or weighting of prediction error responses at early sensory levels. Instead, directing attention toward or away from the omission modality strongly determined the presence and amplitude of higher‐level updating processes associated with phasic LC–NE activity. This suggests an attentional system capable of influencing distinct stages in the processing hierarchy and carries implications for understanding (o)P3 elicitation and its role in learning. Methodologically, the study provides a first step in examining voluntary attention in omission responses while controlling for prediction across conditions. Finally, present results emphasize the importance of keeping prediction constant when investigating attentional effects.

## Author Contributions

**Tjerk T. Dercksen:** conceptualization, data curation, formal analysis, funding acquisition, investigation, methodology, project administration, software, validation, visualization, writing – original draft, writing – review and editing. **Andreas Widmann:** conceptualization, methodology, software, writing – review and editing. **Nicole Wetzel:** conceptualization, methodology, project administration, resources, supervision, writing – review and editing.

## Funding

This work was supported by the Deutsche Forschungsgemeinschaft (DFG DE4080/2‐1).

## Data Availability

The data that support the findings of this study are openly available in OSF at https://osf.io/xprbk, reference number https://doi.org/10.17605/OSF.IO/XPRBK.
